# Round the Hospitals

**Published:** 1920-01-24

**Authors:** 


### ROUND THE HOSPITALS.

A FINAL list was published in the *Gazette* of January 19 containing the names of ladies to whom the King has awarded the Royal Red Cross in recognition of valuable nursing services under the British Red Cross Society and the Order of St. John of Jerusalem. A list of names will appear in our next issue.

THE names of the following ladies have been brought to the notice of the Secretary for War for valuable services rendered on hospital ships during the war:—Sister M. A. Brown, Q.A.I.M.N.S.; Sister A. Dodd and Miss K. Mann, R.R.C., matron, T.F.N.S. The names of the following ladies are to be added to those brought to notice for

**ROUND THE HOSPITALS—(*continued*).**

distinguished and gallant services and devotion to duty in France by Field-Marshal Sir Douglas Haig in his despatch of March 16, 1919:—Mrs. E. McDowell, staff-nurse 10th General Hospital; Miss E. Davies, Mrs. A. Evans, Miss D. M. Nicholls, and Miss A. E. Taylor, V.A.D. The names of the following ladies are to be added to those brought to notice for distinguished and gallant services in Egypt by General Sir E. H. H. Allenby in his despatch of March 5, 1919:—Staff-nurse A. Begg, Sister A. Burnet, Sister E. Gallacher, Staff-nurse S. B. Hughes, Staff-nurse B. Randall, Sister N. Roth, T.F.N.S., Miss A. E. Bestwick, Miss A. Bowman, Miss L. Dove, Miss E. M. Fry, Miss M. D. Moss-Flower; Miss L. M. Quarmby, and Miss C. Rait, V.A.D. The names of the following ladies are to be added to those brought to notice for distinguished and gallant services and devotion to duty in Italy by General F. R. Earl of Cavan in his despatch of January 18, 1919:—Assistant-Matron Miss M. R. Casswell, A.R.R.C., Q.A.I.M.N.S., attached 1/3 (S. Mid.) Field Ambulance, R.A.M.C. (T.F.); Staff-nurse G. Anderson; Sister R. Freeman, Q.A.I.M.N.S.R.; Sister M. Vernon; Assistant-Matron J. Willens, T.F.N.S.; Miss A. Agnew; Miss S. Buxton, head-cook; Miss N. Hawthorne, quartermaster; Miss M. S. Hilditch, laboratory assistant, all of the 38th Stationary Hospital, V.A.D. The name of Sister E. Cooke, Q.A.I.M.N.S., has been added to the list of mentions by Lieut.-General W. R. Marshall for distinguished and gallant services and devotion to duty in Mesopotamia.

The College of Nursing can be warmly congratulated upon the strongly representative character of what was probably the largest gathering of trained nurses, which assembled at the Large Hall of the Automobile Club in Pall Mall. Sir Arthur Stanley was in the Chair, and amongst the speakers were Dr. Addison, Minister of Health; Viscount Sandhurst, who was responsible for the Registration Bill in the House of Lords; Miss Sparshott, and Miss Cox-Davies. The ladies acquitted themselves admirably and their speeches were short, interesting, and to the point. Amongst those present was Sir Robert Morant, to whose services in the passing of the Nurses' Registration Bill Dr. Addison paid a well-deserved and well-earned tribute. The nursing profession now includes many dames, and many of the great ones amongst them were present. There were also several lifelong workers, including Miss Florence Paget, a host in herself, most of the principal matrons, and other notables, many of whom had travelled long distances to be present. All types of nurses were there, keen, alert, interested, full of zeal for their profession, and rejoicing in the splendid opportunities which were now opening for every trained nurse who was equipped to take an active part in its development.

This "At Home" given by Sir Arthur Stanley and other members of the Council of the College at the Royal Automobile Club to celebrate the passing into law of the Nurses' Registration Acts was a notable event in nursing history. As the guests of honour Lord Sandhurst and Dr. Addison were recipients of a resolution of thanks for their services in connection with the passage of the English Bill through Parliament. Dr. Addison delighted the company by again insisting on the importance of paying better salaries to members of the nursing services. He exposed the evils of "the system under which nurses worked very hard and received the wages of a scullery-maid." He pressed for the extension of facilities for good training, and made it clear that he realises the existing defect of the training-schools in failing to prepare nurses for their duties in connection with a widespread service of preventive medicine. His adjuration to nurses to be citizens first and nurses second is in entire accord with the new spirit of revolt against the limitations of the nurses' interests during training to such as lie within the hospital gates.

On the thrilling subject of the first Council Dr. Addison showed that he knew how to hold his tongue. He made the interesting announcement that the chosen band had probably by that time received their invitations, and stirred the hopes of his hearers by laying stress on the responsibility which would rest on the Council for developing a "real professional spirit and professional organisation, sane and well disciplined, working towards good practice and good ideals." Nothing is more certain than that the chief requisite in all the councillors is a sane, well-balanced attitude towards the problems which confront them. Nursing administration is not a political game. It must at all costs be kept out of the arena of heated controversies, and reserved for settlement by present-day, modern nurses who understand their business.

An interesting ceremony, which took place on January 12, marked the erection of the memorial to Miss Edith Cavell, now rapidly approaching completion opposite St. Martin's Church. It will be remembered that the memorial is the outcome of a movement promoted by the *Daily Telegraph.* Viscount Burnham, as proprietor of this paper, and Sir George Frampton, the sculptor, were entrusted with the duty of depositing in a cavity of the structure a leaden box containing documents relating to Nurse Cavell. The casket, which bears the head of a lion, was the gift of the Worshipful Company of Carpenters. It contains the signatures of the King and Queen on vellum, and those of the King and Queen of Belgium, a complete list of the subscribers, and copies of the *Daily Telegraph* describing the events which led to the erection of the monument. The main structure of granite is already in place, and it is possible to form some conception of the great addition to the monumental treasures of London which the finished work will afford.

The case of the disabled war nurse, lately referred to the Ministry of Labour, is receiving at last every attention. There are about 500

**ROUND THE HOSPITALS**—(*continued*).

altogether, and among the 1,400 nurses still to be demobilised there may yet prove to be others. Training is offered to nurses incapacitated from following their profession in any case where they are in receipt of an official pension and are certified by the medical officer of the Ministry of Pensions as fit to undertake the chosen occupation. The injuries which have in most cases made nursing impossible are not wounds, but such after-effects as shell-shock, neurasthenia, heart trouble, or muscular weakness. Some disabled nurses have chosen the allied callings of massage, dispensing, medical electricity, infant-welfare work, and health visiting. Others prefer an occupation entirely dissociated from their former one. But an effort is made in every case to help the disabled nurse to find an opening suited to her tastes and capacity.

---

THE death of Miss Florence Nightingale Shore without return of consciousness in the Sussex County Hospital four days after the assault was committed on her has caused great sorrow among all who knew her, and something like dismay in the public. Miss Shore was the god-child and cousin of Florence Nightingale, and imbibed her vocation for nursing from the fountain-head. The original name of the Nightingale family, it will be remembered, was Shore, and was changed when property was bequeathed to them. Sister Florence Nightingale Shore was trained at the Edinburgh Royal Infirmary, and served through the South African War. She volunteered again for service in 1914, though her age was then forty-five, and worked first under the French Red Cross and then in the Q.A.I.M.N.S.R. She distinguished herself by her gallantry in sticking to her post on duty during a bad air raid at Wimereux, when the nurses were ordered to take shelter, and has given continual evidence of the reality of her vocation. Her brother is Brigadier-General Shore, C.B., D.S.O., who rendered distinguished service in Mesopotamia during the war, and is now in California. The Baroness Farina, whom Miss Shore visited the day before her fatal journey, is her aunt. Miss Shore was staying at a well-known nurses' home in Hammersmith prior to her departure for Hastings, and the matron, Miss Rogers, who was an old friend of hers, saw her off from Victoria when the young man believed to be the murderer got into the train. At the inquest, held on January 19, and adjourned to February 4, the coroner spoke of Miss Shore's life as one of great abnegation—a noble woman whom we could ill afford to lose.

---

THE design for the Victory Medal has been finally approved. On the obverse there is a winged figure of Victory, full length and full face, and on the reverse the words "The Great War for Civilisation." It is identical in its main features with that to be issued by the other Allied and Associated Powers for services in various theatres of war, but differs in its specific treatment of the subject. The Committee which selected it comprised representatives of the Royal Academy, the Royal Society of British Sculptors, the British Museum, the National Gallery, the Victoria and Albert Museum, and the Royal Mint.

---

COMBINED with the bronze oak leaf which the King has approved for members of the British, Dominion, Colonial, and Indian Expeditionary Forces who were happy enough to be Mentioned in Despatches by a commander in the field the Victory ribbon and medal will be a "decoration" worthy of the name. Two emblems are to be provided, one for dress and one for undress wear. Demobilised nurses whose names have been mentioned should apply for the bronze oak leaf to the Secretary, War Office [AG 10], 27 Pilgrim Street, E.C. 4, giving the date of the *Gazette* in which their mention appeared and the locality in which they were serving. There is little doubt the Oak Leaf will be more highly prized than the certificates already issued to those Mentioned in Despatches.

---

WOMEN have not hitherto taken a leading part in scientific research. They are ineligible for research scholarships, and have for the most part been unable, unassisted, to devote many years of their lives to unremunerative labours. We rejoice to see that the sum of £10,000 has lately been given to Girton College by an anonymous friend, to be applied, both principal and interest, during the next twenty years, for the encouragement of research by women in mathematical, physical, and natural sciences. It is intended especially to assist in the development of scientific research as it affects industrial progress. We venture to express the hope that the fund may be further augmented by women interested in the progress made by their *confrères* in this direction, and that it may in this way become a permanent addition to the endowments of Girton.

---

A REUNION dinner of all associated with the Duchess of Westminster's famous hospital at Le Touquet is to take place on January 26 at the Holborn Restaurant, and promises to be a very notable affair. Many distinguished people were connected with it during its five years of good work, some as patients, some as staff. Dinners of this description are very popular at the moment, and serve to bring together old comrades who in the ordinary course of events would never meet again.

---

THE City of London Maternity Hospital is appealing for £10,000 to enable it to be brought up to the standard of modern requirements, and further sums are needed to provide a convalescent home for mothers whose health is impaired. Its record of 8,655 babies born during the war is admirable, especially when it is remembered that refuge had

**ROUND THE HOSPITALS**—(*continued*).

to be frequently sought in the cellars at critical moments during the air raids. The hospital accommodates thirty-six midwifery pupils, and the combined requirements of the training-school and the patients render it highly important that the lighting and heating, the hot-water supply and the offices, shall be of the best. This is not a time when the nation can afford to let old and tried maternity institutions fall behind in the race for perfection, and we trust the required sums will shortly be forthcoming.

---

A pleasant ceremony of congratulation took place at the meeting of the Greenwich Guardians on the announcement of the very satisfactory results obtained in the examination of probationers. The examinations were carried out by Dr. Herbert French, physician to the King's Household and to Guy's Hospital. Dr. French gave a very satisfactory report, and especially mentioned the good quality of the bandaging among the senior nurses. Three of the senior nurses were graded as "excellent," and three as "good." They were introduced to the Chairman of the Board by the matron, Miss Ward, and received his congratulations. The managers are bent on bringing the nursing in the institution up to the level of that in the London voluntary hospitals, and are going the right way to work.

---

Scotland will not be far behind England in constituting the first Council under the new Registration Act. According to the proviso of the Act, the fifteen persons who are to constitute the Council have to be appointed by the Scottish Board of Health. Four of these are to be appointed after consultation with persons and bodies having special knowledge and experience of training-schools for nurses, of the work of matrons of hospitals, of general and special nursing services, and of general and special medical practice. The nine other persons whom the Board of Health has the onus of selecting must be, or must have been at some time, nurses actually engaged in rendering services in direct connection with the nursing of the sick. The Board invites suggestions from nurses and others for filling these places on the Council, being anxious to make it thoroughly representative. All who desire to make recommendations must forward a clear statement of the special qualifications and experience of the persons they propose as members of Council.

---

Miss Charlotte Aikens continues her lively and instructive articles on South American hospitals in the December number of *The Trained Nurse* (New York). Bolivia is the subject, and we learn some curious facts about the ancient hospital at La Paz, with accommodation for 500 male patients. "In the ward which I visited there were ninety beds radiating from the centre in four wings . . . every bed seemed to be occupied, but no nurse or attendant was in sight. This was typical of nearly all the South American hospitals, with the exception of those seen in Buenos Aires. No one ever seemed to be doing anything for the patients. Across from the entrance to this hospital was a coffin factory, ready to supply coffins for all. I was told that six hearses formed a part of the hospital equipment, but there was no ambulance." Trained nurses are evidently the great need, and in this respect Bolivia has all to do.

---

Reports come through to the *Times* of a new outbreak of typhus in Middle Europe, coupled with smallpox and plague. Typhus is already raging in Eastern Galicia and particularly in Cracow, while plague has made its appearance at Chocim on the Dniester. The epidemic is indubitably being carried far and wide by refugees from Russia, where 1,340,000 cases of typhus were reported from September 1918 to March 1919, and since the latter date it is estimated there have been half as many more again. The break-up of Deniken's and Petlura's armies led to huge straggling bodies of men, sixty per cent of whom were infected, deserting towards Poland. The conditions are inconceivably wretched for those attacked by disease. The doctors are too few in number to be of any use, and they have no medical stores. There are no nurses as there is no accommodation for them. Soap, linen, beds, and disinfectants are the most urgent needs. Colonel Gilchrist, with the American Typhus Fighting Commission, and a British detachment from the Society of Friends are accomplishing wonders with a very slender personnel. They need reinforcements at every point, and particularly well-seasoned and experienced workers.

---

An "alleged bogus nurse" has been convicted of theft and sentenced at Brighton to two months' hard labour. She was dressed as a nurse, told stories of her experiences during the war, and was wearing the 1914 ribbon with other war decorations. With these credentials she succeeded in stealing a gold-watch bracelet, valued at £6. No trace of her ever having connection with the Army nursing service has come to light. Will registration deliver the public from this kind of thing? If it makes women suspicious of casual nurses who do not wear the registered uniform it will do some good. But what impostor ever lacks a plausible excuse?

---

With a view to providing a permanent maternity hospital for lying-in women, the Leicester Corporation have secured a large house and grounds at a cost of £8,500. It is estimated that the building will be capable of accommodation of thirty patients at a time. The sanction of the Ministry of Health is being sought. Temporary provision has been made for sixteen patients in a large house in Victoria Road by arrangement with the Leicester Health Society.

